# Electrospun carbon nanofibers reinforced 3D porous carbon polyhedra network derived from metal-organic frameworks for capacitive deionization

**DOI:** 10.1038/srep32784

**Published:** 2016-09-09

**Authors:** Yong Liu, Jiaqi Ma, Ting Lu, Likun Pan

**Affiliations:** 1 Engineering Research Center for Nanophotonics & Advanced Instrument, Ministry of Education, School of Physics and Material Science, East China Normal University, Shanghai 200062, China; 2 Chemistry Department, Soochow University, Suzhou 215123, China

## Abstract

Carbon nanofibers reinforced 3D porous carbon polyhedra network (e-CNF-PCP) was prepared through electrospinning and subsequent thermal treatment. The morphology, structure and electrochemical performance of the e-CNF-PCP were characterized using scanning electron microscopy, Raman spectra, nitrogen adsorption-desorption, cyclic voltammetry and electrochemical impedance spectroscopy, and their electrosorption performance in NaCl solution was studied. The results show that the e-CNF-PCP exhibits a high electrosorption capacity of 16.98 mg g^−1^ at 1.2 V in 500 mg l^−1^ NaCl solution, which shows great improvement compared with those of electrospun carbon nanofibers and porous carbon polyhedra. The e-CNF-PCP should be a very promising candidate as electrode material for CDI applications.

With an increasing human population combined with the over-exploitation of water resources for industrial and agricultural activities, water shortage has become the most serious problem in the 21th century^1^,^2^. Although there exist some approaches, including economic and recycling use of water for human and animal consumption that can mitigate the problem to some extent, alternative sources of freshwater are required to meet its growing need^3^. As most of earth’s water is saline water which is not suitable for direct consumption, desalination has emerged to be the most promising approach to generate freshwater. Conventional desalination techniques such as membrane separation and flash distillation require either high-pressure pumps, membranes or thermal heaters, which will result in high capital or operational expenditure^4^. Therefore, it is in great urgency to find a low-cost yet highly-efficient desalination technology.

Capacitive deionization (CDI), also known as electrosorption, has entered in a rapid development period in the last ten years due to its minimum energy consumption and environmental friendliness compared to traditional desalination techniques^5^,^6^,^7^,^8^,^9^,^10^,^11^,^12^,^13^,^14^. This emerging technology is proceeded by adsorbing ions into the double layer formed at the electrode surface when a low direct current potential (normally less than 2 V) is applied. In CDI devices, the electrode materials are regarded as the most important component because the device efficiency strongly depends upon their properties. Since the pioneering work in the 1960s^15^, various carbonaceous materials including activated carbon (AC)^16^,^17^, carbon nanofibers (CNFs),^18^,^19^ carbon aerogels (CAs),^20^ mesoporous carbon (MC)^21^,^22^, carbon nanotubes (CNTs)^23^ and graphene^24^,^25^,^26^ have been used as CDI electrodes. However, these carbon materials suffer some problems such as high manufacturing cost and low adsorption capacity which limit CDI from being used for scaling-up application^27^. Therefore, exploring new strategies for carbonaceous material fabrication is necessary for current CDI application.

Recently, direct carbonization of metal-organic frameworks (MOFs) has been demonstrated to be a facile route to synthesize porous carbon materials^28^,^29^ with extremely high surface area which have shown some appealing performances in energy storage device such as lithium/sodium ion battery^30^ and supercapacitor^31^,^32^,^33^. In our previous work^34^, MOFs-derived porous carbon polyhedra (PCP) were studied as electrode material for CDI and they exhibited excellent performance with an electrosorption capacity (EC) of 13.86 mg g^−1^ in 500 mg l^−1^ NaCl solution. However, there are still some serious problems faced by the PCP electrode in previous work: (i) the surface pores (micropores, mesopores or macropores generated from the gas release during the pyrolysis process, which is the most critical factor for the electrosorption process) of PCP are mainly distributed in micropore range, which may cause the electrical double layer overlapping effect to hinder the CDI performance; (ii) the use of binder will increase the internal resistance and block some of the pores in the carbon materials, resulting in lower adsorption capacity.

Electrospinning, as a simple yet effective technique to fabricate continuous CNFs, has attracted much attention in the fields like energy storage^35^,^36^ and CDI^18^,^19^,^37^,^38^. Particularly, the electrospinning of polyacrylonitrile (PAN) precursor followed by carbonization can get free-standing CNFs with ultra-fine and controllable diameter^18^,^19^. And also the electrospun CNFs (e-CNFs) contain a large portion of mesopores and macropores^37^,^39^. Thus, an obvious expectation can be made that a combination of free-standing e-CNFs and PCP with hierarchical pore structure should be a promising electrode material for CDI application. Unfortunately, such an exploration has not been reported in the literatures so far.

In this work, CNFs reinforced 3D PCP network (e-CNF-PCP) was fabricated through electrospinning followed by thermal treatment and used as electrode materials for CDI for the first time. The e-CNF-PCP exhibits excellent performance with an EC of 16.98 mg g^−1^ in 500 mg l^−1^ NaCl solution, which shows great improvement compared to those of PCP and e-CNFs.

## Result and Discussion

**Figure 1:**
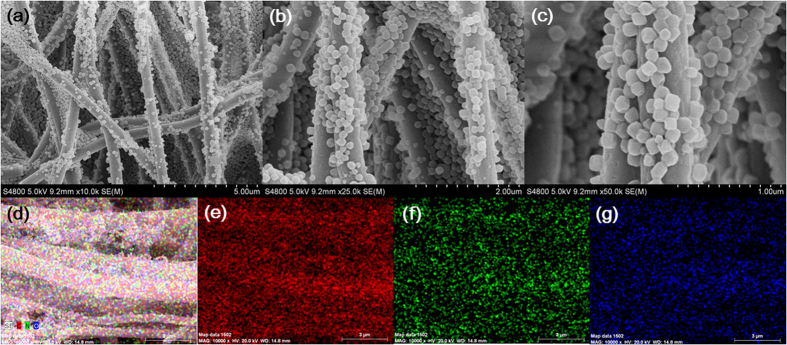
(**a**–**c**) FESEM images of e-CNF-PCP at different magnifications and elemental mapping images of (**d**) e-CNF-PCP, (**e**) C element, (**f**) N element and (**g**) O element.

Figure 1(a–c) present the scanning electron microscopy (FESEM) images of e-CNF-PCP at different magnifications. It can be observed that the polyhedra structure of the MOFs derived PCP is perfectly maintained and homogeneously covered on the surface of e-CNFs. The e-CNF-PCP displays a three-dimensional (3D) framework structure consisting of carbonaceous particles. Such a highly porous network structure provides more tunnels for penetration by solutions and allows easy access by ions. Figure 1(d) shows the energy dispersive X-ray spectroscopy (EDS) mapping image of e-CNF-PCP and Fig. 1(e–g) show the distributions of C, N and O elements, respectively. It can be seen that the C, N and O elements are homogeneously distributed throughout the e-CNF-PCP.

**Figure 2:**
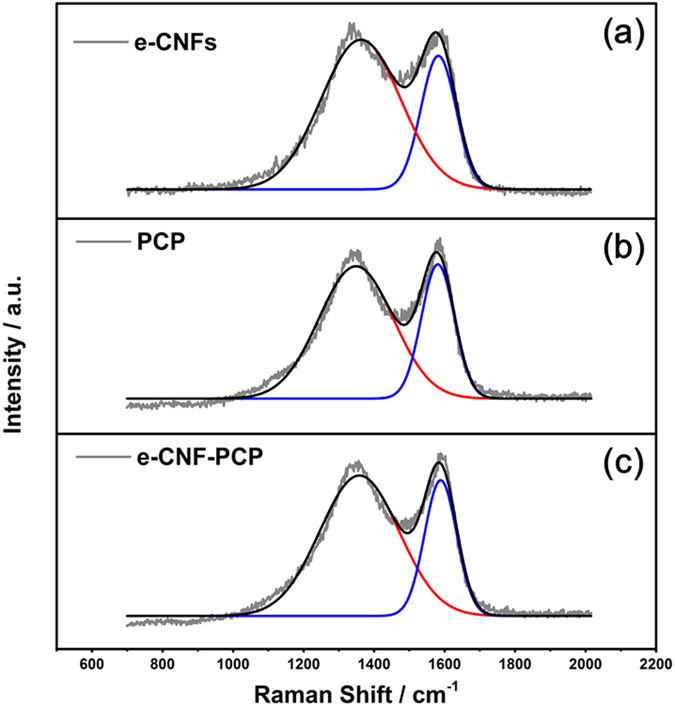
Raman spectra of (**a**) e-CNFs, (**b**) PCP and (**c**) e-CNF-PCP.

Figure 2(a–c) show Raman spectra of e-CNFs, PCP and e-CNF-PCP. The Raman spectra display two peaks at 1344 and 1590 cm^−1^, corresponding to D line and G line, respectively, which are signatures of graphitic carbon materials. The G line corresponds to the crystalline graphitic carbon and the D line is attributed to the defect and disordered structure. It is generally accepted that the relative intensity ratio of D peak to G peak (I_D_/I_G_) is the indication of disorder or defect in the carbon structure^40^,^41^. The I_D_/I_G_ values of e-CNFs, PCP and e-CNF-PCP are 0.89, 0.99 and 1.04, respectively, indicating that the synergistic effect of e-CNFs and PCP generates more defects in the e-CNF-PCP. The presence of defects can generate more accessible surface area and cause an increase in ability for the accumulation of charges, which is beneficial to the charge transfer in the adsorption process^42^,^43^.

**Figure 3:**
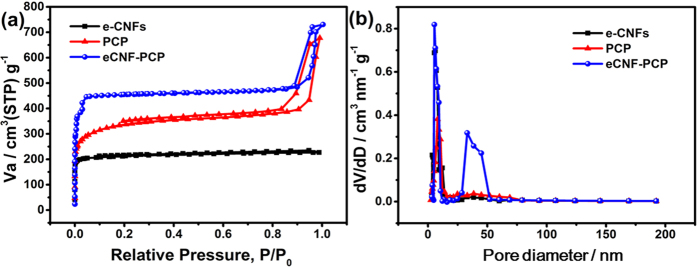
(**a**) Nitrogen adsorption-desorption isotherms and (**b**) pore size distribution of e-CNFs, PCP and e-CNF-PCP.

It is universally known that large accessible surface area and suitable pore size are crucial for the capacitive performance. Therefore, nitrogen adsorption-desorption and pore size distribution analysis of e-CNFs, PCP and e-CNF-PCP were performed and the results are presented in Fig. 3(a,b). It is clearly observed that all isotherms show typical type IV behaviour. The specific surface area, pore volume and mean pore diameter were determined by the Brunauer-Emmett-Teller method and are listed in Table 1. It can be seen that the e-CNF-PCP has a largest specific surface area of 1450.6 m^2^ g^−1^. Clearly, the as-synthesized e-CNF-PCP displays a highly porous structure. The pore size distribution reveals that the e-CNFs and PCP are mainly composed of micropores and mesopores. However, the e-CNF-PCP exhibits a hierarchical pore structure consisting of not only micropores and mesopores but also macropores that are generated from the interconnected open channels between the nanofibers and the PCP. This 3D hierarchical pore structure can lower the resistance and shorten the ion transport pathway, which is beneficial to the electrosorption process.

**Figure 4:**
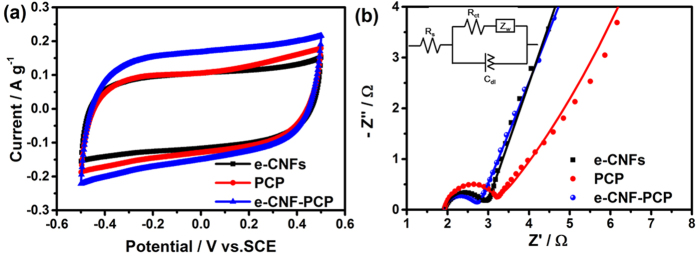
(**a**) CV curves and (**b**) Nyquist plots of e-CNFs, PCP and e-CNF-PCP in 1 M NaCl solution.

Figure 4a shows the potential sweep cyclic voltammetry (CV) curves of e-CNFs, PCP and e-CNF-PCP electrodes at a potential sweep rate of 1 mV s^−1^ within a potential range of −0.5–0.5 V, respectively. All CV curves are nearly rectangular. The current increases and decreases steadily with the electric potential, indicating that no faradaic reaction happens and ions are adsorbed on the electrode surface by forming an electric double layer. The specific capacitances of e-CNFs, PCP and e-CNF-PCP electrodes are 217.13, 261.33 and 284.75 F g^−1^, respectively, as shown in Table 2. Obviously, the e-CNF-PCP electrode exhibits an improved specific capacitance than PCP and e-CNFs. The reason should be mainly ascribed to the following: (i) the e-CNF-PCP exhibits higher specific surface area than PCP and e-CNFs, which can increase the double layer capacitance; (ii) the 3D hierarchical pore structure of e-CNF-PCP can lower the resistance and shorten the ion transport pathway, which can be proved by the electrochemical impedance spectroscopy (EIS) measurements. Figure 4b shows the Nyquist plots of e-CNFs, PCP and e-CNF-PCP electrodes. The Nyquist plots are fitted and interpreted with the help of an equivalent electric circuit, as shown in the inset of Fig. 4b. The high frequency arc corresponds to the charge transfer limiting process and is ascribed to the double-layer capacitance (C_dl_) in parallel with the charge transfer resistance (R_ct_) at the contact interface between the electrode and the electrolyte solution. The inclined line, resulting from the Warburg impedance (Z_W_), corresponds to the ion-diffusion process in the electrolyte. The fitted R_ct_ values for the electrodes are listed in Table 2. Obviously, the e-CNF-PCP electrode enjoys a much lower R_ct_, which is beneficial to the capacitive performance; (iii) compared to the PCP, the e-CNF-PCP is employed as free-standing electrode without the use of polymer binder.

**Figure 5:**
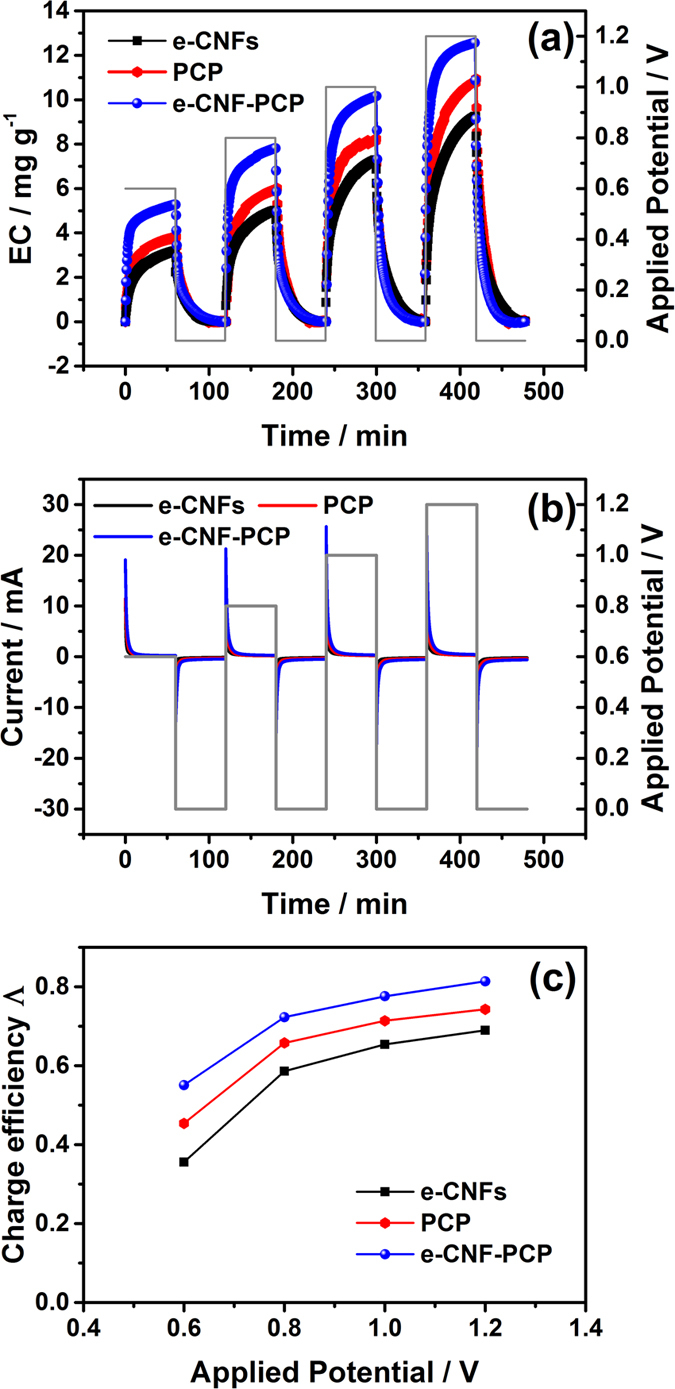
(**a**) EC transient, (**b**) current transient and (**c**) charge efficiency for e-CNFs, PCP and e-CNF-PCP electrodes at different applied potentials.

Figure 5(a,b) show the EC transients and corresponding currents for e-CNFs, PCP and e-CNF-PCP electrodes in NaCl solution with an initial concentration of ~300 mg l^−1^ during batch-mode experiments at four different applied voltages: 0.6, 0.8, 1.0 and 1.2 V, respectively. All the charge processes were recorded until sorption equilibrium reached, and then the electrodes were shorted for regeneration. As expected, the ECs of these electrodes increase dramatically once the cell voltage is applied. High voltage results in high salt removal because of enhanced electrostatic force. The equilibrium ECs of e-CNFs, PCP and e-CNF-PCP electrodes at different voltages are listed in Table 3. As expected, the e-CNF-PCP electrode possesses the best electrosorption performance with an EC of 12.56 mg g^−1^ at 1.2 V, higher than those of e-CNFs (9.25 mg g^−1^) and PCP (10.94 mg g^−1^).

Charge efficiency (Λ) is a functional tool to gain insight into the EDL formed at the interface between the electrode and the solution^27^,^44^,^45^,^46^,^47^, as described according to the following equation:


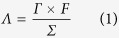


where F is the Faraday constant (96485 C mol^−1^), Γ is the EC (mol g^−1^) and Σ (charge, C g^−1^) is obtained by integrating the corresponding current. The charge efficiencies of e-CNFs, PCP and e-CNF-PCP electrodes at 0.8–1.2 V are shown in Fig. 5(c) and Table 3. It can be seen that all electrodes show a similar relation between Λ and cell voltage: Λ increases with the increase of the applied voltage, which is consistent to that reported in the literature^48^. Most importantly, the Λ of e-CNF-PCP shows an obvious improvement compared to those of e-CNFs and PCP, which is mainly ascribed to its lower charge transfer resistance.

**Figure 6:**
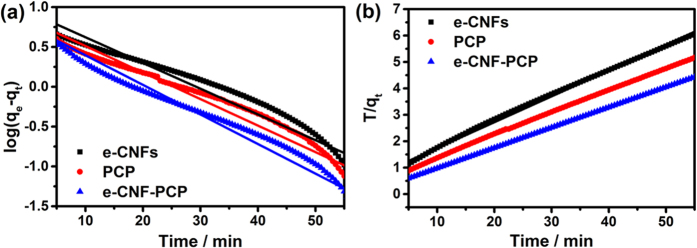
Linear fitting of the electrosorption of NaCl by e-CNFs, PCP and e-CNF-PCP electrodes using (**a**) pseudo-first-order kinetic equation and (**b**) pseudo-second-order kinetic equation.

Adsorption kinetics, indicating the adsorption rate, is an important characteristic of adsorbents. It can be determined by using the pseudo-first-order adsorption kinetics and pseudo-second-order adsorption kinetics equations, which are often presented as:


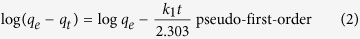



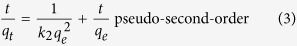


where q_e_ (mg g^−1^) and q_t_ (mg g^−1^) are the amounts of NaCl adsorbed at equilibrium and time t (min), respectively. k_1_ (mg g^−1^ min^−1^) and k_2_ (g mg^−1^ min^−1^) are the adsorption rate constants of pseudo-first-order and pseudo second order equations, respectively. Figure 6 shows the linear fitting between the equations and experimental data. The rate constants as presented in Table 4 canbe obtained from the slopes and intercepts of the fitting lines in Fig. 6. Normally closeness of regression coefficients to 1 supports the assumption of kinetics for the adsorption processes and it is found that pseudo-second-order kinetics equation describes the electrosorption behavior better. The k_2_ values of e-CNFs, PCP and e-CNF-PCP are 0.012, 0.014, 0.027, respectively. The higher rate constant of e-CNF-PCP indicates that its 3D hierarchical pore structure facilitates the quick access of ions onto its surface.

**Figure 7:**
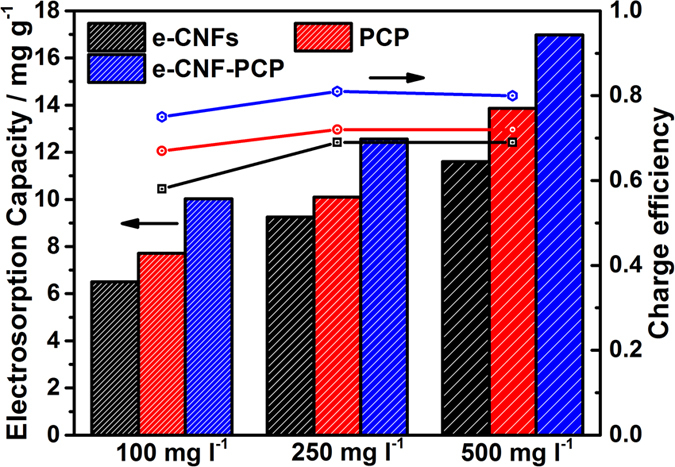
ECs and charge efficiencies of e-CNFs, PCP and e-CNF-PCP in NaCl solutions with different initial concentrations.

To get a better understanding of the electrosorption behavior of e-CNF-PCP, the electrosorption experiments using e-CNF-PCP electrode in NaCl solution with different initial concentrations at 1.2 V were carried out and the ECs and charge efficiencies were compared with those of PCP and e-CNFs, as shown in Fig. 7 (the data of PCP was obtained from our previous work^34^), As seen the ECs/Λ of e-CNF-PCP in 100, 250, 500 mg l^−1^ NaCl solutions are 10.03 mg g^−1^/0.75, 12.56 mg g^−1^/0.81 and 16.98 mg g^−1^/0.80, which are higher than those of PCP (7.71 mg g^−1^/0.67, 10.10 mg g^−1^/0.72 and 13.86 mg g^−1^/0.72) and e-CNFs (6.5 mg g^−1^/0.58, 9.25 mg g^−1^/0.69 and 11.60 mg g^−1^/0.69). Obviously, e-CNF-PCP exhibits an improved CDI performance in all concentrations, which further confirms that e-CNF-PCP is an excellent electrode material for CDI application.

In order to further evaluate the electrosorption performance of e-CNF-PCP, the CDI performance and manufacturing difficulty of e-CNF-PCP is compared with those of other carbon electrode materials reported in the literatures, as shown in Table 5. Obviously, the electrosorption capacity of e-CNF-PCP is among the highest value, which is attributed to its high specific surface area, hierarchical pore structure and excellent charge transfer ability. Also, the e-CNF-PCP is a freestanding electrode, which prevents the manufacturing and industrial difficulties such as electrode coating process and binder issues^18^,^37^.

## Method

### Fabrication

The e-CNFs were prepared in a typical process. 1 g PAN (average MW 150 000, Aldrich) was dissolved in 8.8 g N, N-dimethylformamide to form a homogeneous solution by stirring at 60 °C for 10 h. Then, the polymer solution was transferred into a 10 ml syringe connected to a stainless steel needle. Electrospinning was carried out by applying a positive voltage of 22 kV to the needle via an aluminum collector roller. The distance between the needle tip and the collector was 15 cm and the flow rate of the solution was set at 1 ml h^−1^. The as-collected fibers were stabilized at 280 °C for 2 h in air at a heating rate of 2 °C min^−1^.

The as-prepared e-CNFs was firstly pre-treated using zinc nitrate solution through ultrasonic spray method at a frequency of 1.65 MHz. Then the pre-treated e-CNFs cloth was immersed into a methanol solution containing 2.58 g zinc nitrate and 2.63 g 2-methylimidazole. After agitation the solution was aged at 25 °C for 24 h to get the e-CNFs-ZIF-8. After that, the as-synthesized e-CNFs-ZIF-8 was subsequently thermally treated at 1200 °C in nitrogen for 2 h with a heating rate of 2 °C/min. Finally, the e-CNF-PCP was obtained by washing extensively by HCl to remove residual Zn^2+^.

### Characterization

The surface morphology and structure of the as-synthesized e-CNF-PCP were examined by FESEM (JEOL JSM-LV5610), EDS (Oxford), and Raman spectroscopy (Renishaw inVia). Nitrogen adsorption-desorption isotherms were measured at 77 K with an ASAP 2020 Accelerated Surface Area and Porosimetry System (Micrometitics, Norcross, GA). The CV and EIS measurements were carried out in 1 M NaCl solution by using Autolab PGSTAT 302N electrochemical workstation in a three-electrode mode, including a standard calomel electrode as reference electrode and a platinum foil as counter electrode. In EIS measurement, a frequency range of 0.1 Hz to 100 kHz and an AC amplitude of 5 mV were applied. The specific capacitance (C_sp_ in F g^−1^) can be obtained from the CV process according to the equation:


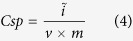


where 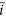 (A) is the average current, v (V s^−1^) is the scan rate and m (g) is the total mass of electrodes.

### Electrosorption experiments

The e-CNF-PCP was used directly as free-standing CDI electrodes. Electrosorption experiments were conducted in a continuously recycling system including a unit cell^49^,^50^,^51^,^52^. In each experiment, the analytical pure NaCl solution was continuously pumped from a peristaltic pump into the cell and the effluent returned to the unit cell with a flow rate around 50 ml min^−1^. The volume and temperature of the solution were maintained at 50 ml and 298 K, respectively. Meanwhile, the variation of NaCl concentration was monitored and measured at the outlet of the unit cell by using a conductivity meter (DDS-308, Precision & Scientific Instrument). The relationship between conductivity and concentration was obtained according to a calibration table made prior to the experiment, which has been described in our previous work^26^.

In our experiment, the EC is defined as follows:


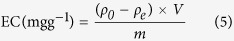


where ρ_0_ (mg l^−1^) and ρ_e_ (mg l^−1^) are initial and final NaCl concentrations, respectively. V (l) is the solution volume, and m (g) is the total mass of the electrodes.

## Additional Information

**How to cite this article**: Liu, Y. *et al*. Electrospun carbon nanofibers reinforced 3D porous carbon polyhedra network derived from metal-organic frameworks for capacitive deionization. *Sci. Rep.* **6**, 32784; doi: 10.1038/srep32784 (2016).

## Figures and Tables

**Table 1:** Specific surface areas, pore volumes and mean pore diameters of e-CNFs, PCP and e-CNF-PCP.

Sample	Specific surface area (m^2^ g^−1^)	Pore volume (cm^3^ g^−1^)	Mean pore diameter (nm)
e-CNFs	937.4	0.70	3.74
PCP	1187.8	0.78	3.50
e-CNF-PCP	1450.6	1.01	3.84

**Table 2:** Electrochemical performances of e-CNFs, PCP and e-CNF-PCP.

	e-CNFs	PCP	e-CNF-PCP
Specific capacitance / F g^−1^	217.13	261.33	284.75
R_ct_/Ω	0.84	0.90	0.70

**Table 3:** The equilibrium ECs and charge efficiencies of e-CNFs, PCP and e-CNF-PCP electrodes at 0.6–1.2 V.

	0.6 V		0.8 V		1.0 V		1.2 V	
	EC (mg g^−1^)	Λ	EC (mg g^−1^)	Λ	EC (mg g^−1^)	Λ	EC (mg g^−1^)	Λ
e-CNFs	3.19	0.36	5.02	0.59	7.29	0.65	9.25	0.69
PCP	3.83	0.45	6.02	0.66	8.24	0.71	10.94	0.74
e-CNF-PCP	5.28	0.55	7.81	0.72	10.16	0.78	12.56	0.81

**Table 4:** Coefficients of kinetic equations for the electrosorption in NaCl solution.

Applied Voltage		e-CNFs	PCP	e-CNF-PCP
Pseudo-first-order kinetic equation	k_1_	0.074	0.077	0.086
	r^2^	0.913	0.933	0.951
Pseudo-second-order kinetic equation	k_2_	0.012	0.014	0.027
	r^2^	0.998	0.999	0.999

**Table 5:** Comparison of CDI performances and manufacturing difficulties among various carbon electrode materials from the literatures.

Electrode material	CDI performancesNaCl concentration(mg l^−1^) EC (mg g^−1^)		Manufacturing difficulties	Ref.
CAs	~500	2.9	Supercritical drying, binder	20
AC	500	9.72	Coating process, binder	45,50
	1000	10.80		
	1500	11.00		
	2000	11.76		
CNTs	500	2.57	Chemical vapor deposition, binder	51,52
	1000	3.71		
	1500	4.76		
	2000	5.24		
e-CNF-PCP	100	10.03	—	(This work)
	300	12.56		
	500	16.98		
